# Supplementary material for: Adapting an Atmospheric Dispersion Model to Assess the Risk of Windborne Transmission of Porcine Reproductive and Respiratory Syndrome Virus between Swine Farms
Source: Viruses. 2022 Jul 28;14(8):1658. doi: 10.3390/v14081658 (PMC9416339; doi:10.3390/v14081658)
Supplement: Supplementary file 1 [file viruses-14-01658-s001.zip › Figure S1.pdf]

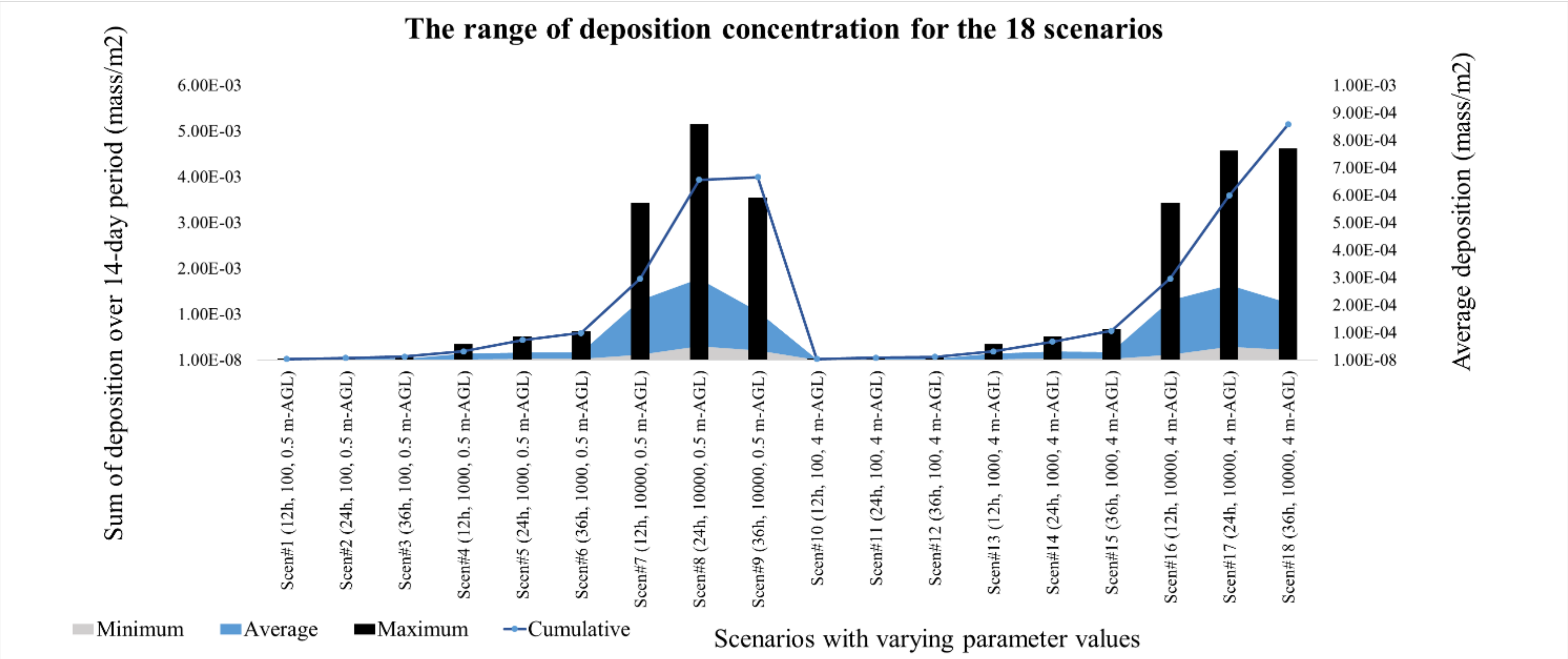

Figure S1. The range of deposition values resulted from the 18 scenarios with independently varying input parameter values. This excludes the emitting farms. The parameters combinations included 1) emission quantity, i.e. the particle concentration at source farms (n=3; 100, 1000, 10000 particles), 2) model duration which is a proxy for pathogen survival (n=3; 12h, 24h, 36h), and 3) takeoff/emission height (n=2; 0.5 and 4 m-AGL). This summarization depicts the optimum parameter combinations that results in highest deposition on the terrain.
