# Supplementary material for: Adapting an Atmospheric Dispersion Model to Assess the Risk of Windborne Transmission of Porcine Reproductive and Respiratory Syndrome Virus between Swine Farms
Source: Viruses. 2022 Jul 28;14(8):1658. doi: 10.3390/v14081658 (PMC9416339; doi:10.3390/v14081658)
Supplement: Supplementary file 1 [file viruses-14-01658-s001.zip › Table S1.pdf]

Table S1. References used in the systematic review process to identify parameters relevant to Atmospheric Dispersion Modeling of PRRSv.

1. Alonso C, Davies PR, Polson DD, Dee SA, Lazarus WF. Financial implications of installing air filtration systems to prevent PRRSV infection in large sow herds. Preventive Veterinary Medicine. 2013;111(3-4):268-77. doi: 10.1016/j.prevetmed.2013.05.001. PubMed PMID: WOS:000322297500010.
2. Alonso C, Murtaugh MP, Dee SA, Davies PR. Epidemiological study of air filtration systems for preventing PRRSV infection in large sow herds. Preventive Veterinary Medicine. 2013;112(1-2):109-17. doi: 10.1016/j.prevetmed.2013.06.001. PubMed PMID: WOS:000324155600011.
3. Alonso C, Olson BA, Goyal S, Raynor PC, Davies PR, Torremorell M. Comparison of two size-differentiating air samplers for detecting airborne swine viruses under experimental conditions. Aerosol Science and Technology. 2017;51(2):198-205. doi: 10.1080/02786826.2016.1249278. PubMed PMID: WOS:000394661600008.
4. Alonso C, Otake S, Davies P, Dee S. An evaluation of interventions for reducing the risk of PRRSV introduction to filtered farms via retrograde air movement through idle fans. Veterinary Microbiology. 2012;157(3-4):304-10. doi: 10.1016/j.vetmic.2012.01.010. PubMed PMID: WOS:000304643800006.
5. Alonso C, Raynor PC, Davies PR, Morrison RB, Torremorell M. Evaluation of an electrostatic particle ionization technology for decreasing airborne pathogens in pigs. Aerobiologia. 2016;32(3):405-19. doi: 10.1007/s10453-015-9413-3. PubMed PMID: WOS:000382136700004.
6. Alonso C, Raynor PC, Davies PR, Torremorell M. Concentration, Size Distribution, and Infectivity of Airborne Particles Carrying Swine Viruses. Plos One. 2015;10(8):12. doi: 10.1371/journal.pone.0135675. PubMed PMID: WOS:000360018600063.
7. Alonso C, Raynor PC, Goyal S, Olson BA, Alba A, Davies PR, et al. Assessment of air sampling methods and size distribution of virus-laden aerosols in outbreaks in swine and

poultry farms. Journal of Veterinary Diagnostic Investigation. 2017;29(3):298-304. doi: 10.1177/1040638717700221. PubMed PMID: WOS:000400122500005.

8. Amirpour Haredasht S, Polson D, Main R, Lee K, Holtkamp D, Martínez-López B. Modeling the spatio-temporal dynamics of porcine reproductive and respiratory syndrome cases at farm level using geographical distance and pig trade network matrices. BMC Veterinary Research. 2017;13(1). doi: 10.1186/s12917-017-1076-6.
9. Arruda AG, Sanhueza J, Corzo C, Vilalta C. Assessment of area spread of porcine reproductive and respiratory syndrome (PRRS) virus in three clusters of swine farms. Transboundary and Emerging Diseases. 2018;65(5):1282-9. doi: 10.1111/tbed.12875.
10. Arruda AG, Tousignant S, Sanhueza J, Vilalta C, Poljak Z, Torremorell M, et al. Aerosol Detection and Transmission of Porcine Reproductive and Respiratory Syndrome Virus (PRRSV): What Is the Evidence, and What Are the Knowledge Gaps? Viruses-Basel. 2019;11(8):17. doi: 10.3390/v11080712. PubMed PMID: WOS:000482993900078.
11. Arruda AG, Vilalta C, Perez A, Morrison R. Land altitude, slope, and coverage as risk factors for Porcine Reproductive and Respiratory Syndrome (PRRS) outbreaks in the United States. PLoS ONE. 2017;12(4). doi: 10.1371/journal.pone.0172638.
12. Brito B, Dee S, Wayne S, Alvarez J, Perez A. Genetic Diversity of PRRS Virus Collected from Air Samples in Four Different Regions of Concentrated Swine Production during a High Incidence Season. Viruses-Basel. 2014;6(11):4424-36. doi: 10.3390/v6114424. PubMed PMID: WOS:000345565200019.
13. Brockmeier SL, Lager KM. Experimental airborne transmission of porcine reproductive and respiratory syndrome virus and Bordetella bronchiseptica. Veterinary Microbiology. 2002;89(4):267-75. doi: 10.1016/s0378-1135(02)00204-3. PubMed PMID: WOS:000178803400002.
14. Brookes VJ, Hernandez-Jover M, Holyoake P, Ward MP. Import risk assessment incorporating a dose-response model: Introduction of highly pathogenic porcine reproductive and respiratory syndrome into Australia via illegally imported raw pork. Preventive Veterinary Medicine. 2014;113(4):565-79. doi: 10.1016/j.prevetmed.2014.01.016. PubMed PMID: WOS:000332810300022.

15. Busse FW, Alt M, Janthur I, Neumann W, Schoss P. EPIDEMIOLOGIC STUDIES ON PORCINE REPRODUCTIVE AND RESPIRATORY SYNDROME (PRRS). Tieraerztliche Umschau. 1991;46(12):708-+. PubMed PMID: WOS:A1991GT99800002.
16. Cho JG, Dee SA. Porcine reproductive and respiratory syndrome virus. Theriogenology. 2006;66(3):655-62. doi: 10.1016/j.theriogenology.2006.04.024. PubMed PMID: WOS:000239522000023.
17. Cho JG, Deen J, Dee SA. Influence of isolate pathogenicity on the aerosol transmission of Porcine reproductive and respiratory syndrome virus. Canadian Journal of Veterinary Research-Revue Canadienne De Recherche Veterinaire. 2007;71(1):23-7. PubMed PMID: WOS:000242697500004.
18. Clack HL, Xia T, Lin Z, Lee EM, Melotti K, Rohde M, et al., editors. Non-thermal plasma inactivation of porcine reproductive and respiratory syndrome (PRRS) virus in HOG barn ventilation air. Proceedings of the Air and Waste Management Association's Annual Conference and Exhibition, AWMA; 2019.
19. Corn JL, Cumbee JC, Barfoot R, Erickson GA. Pathogen exposure in feral swine populations geographically associated with high densities of transitional swine premises and commercial swine production. Journal of Wildlife Diseases. 2009;45(3):713-21. doi: 10.7589/0090-3558-45.3.713.
20. Cutler TD, Wang C, Hoff SJ, Kittawornrat A, Zimmerman JJ. Median infectious dose (ID50) of porcine reproductive and respiratory syndrome virus isolate MN-184 via aerosol exposure. Veterinary Microbiology. 2011;151(3-4):229-37. doi: 10.1016/j.vetmic.2011.03.003.
21. Cutler TD, Wang C, Hoff SJ, Zimmerman JJ. Effect of temperature and relative humidity on ultraviolet (UN254) inactivation of airborne porcine respiratory and reproductive syndrome virus. Veterinary Microbiology. 2012;159(1-2):47-52. doi: 10.1016/j.vetmic.2012.03.044. PubMed PMID: WOS:000308121500006.
22. Dee SA, Batista L, Deen J, Pijoan C. Evaluation of systems for reducing the transmission of Porcine reproductive and respiratory syndrome virus by aerosol. Canadian Journal of

Veterinary Research-Revue Canadienne De Recherche Veterinaire. 2006;70(1):28-33. PubMed PMID: WOS:000235347100004.

23. Dee SA, Deen J, Cano JP, Batista L, Pijoan C. Further evaluation of alternative air-filtration systems for reducing the transmission of Porcine reproductive and respiratory syndrome virus by aerosol. Canadian Journal of Veterinary Research-Revue Canadienne De Recherche Veterinaire. 2006;70(3):168-75. PubMed PMID: WOS:000238738000002.
24. Dee SA, Deen J, Jacobson L, Rossow KD, Mahlum C, Pijoan C. Laboratory model to evaluate the role of aerosols in the transport of porcine reproductive and respiratory syndrome virus. Veterinary Record. 2005;156(16):501-4. PubMed PMID: WOS:000228839200015.
25. Dee S, Batista L, Deen J, Pijoan C. Evaluation of an air-filtration system for preventing aerosol transmission of Porcine reproductive and respiratory syndrome virus. Canadian Journal of Veterinary Research-Revue Canadienne De Recherche Veterinaire. 2005;69(4):293-8. PubMed PMID: WOS:000233175400009.
26. Dee S, Cano JP, Spronk G, Reicks D, Ruen P, Pitkin A, et al. Evaluation of the Long-Term Effect of Air Filtration on the Occurrence of New PRRSV Infections in Large Breeding Herds in Swine-Dense Regions. Viruses-Basel. 2012;4(5):654-62. doi: 10.3390/v4050654. PubMed PMID: WOS:000305801700001.
27. Dee S, Otake S, Deen J. Use of a production region model to assess the efficacy of various air filtration systems for preventing airborne transmission of porcine reproductive and respiratory syndrome virus and Mycoplasma hyopneumoniae: Results from a 2-year study. Virus Research. 2010;154(1-2):177-84. doi: 10.1016/j.virusres.2010.07.022. PubMed PMID: WOS:000285124600019.
28. Dee S, Otake S, Oliveira S, Deen J. Evidence of long distance airborne transport of porcine reproductive and respiratory syndrome virus and Mycoplasma hyopneumoniae. Veterinary Research. 2009;40(4):13. doi: 10.1051/vetres/2009022. PubMed PMID: WOS:000267963400009.
29. Dee S, Pitkin A, Deen J. Evaluation of alternative strategies to MERV 16-based air filtration systems for reduction of the risk of airborne spread of porcine reproductive

and respiratory syndrome virus. Veterinary Microbiology. 2009;138(1-2):106-13. doi: 10.1016/j.vetmic.2009.03.019. PubMed PMID: WOS:000267778100014.

30. Dee S, Pitkin A, Otake S, Deen J. A four-year summary of air filtration system efficacy for preventing airborne spread of porcine reproductive and respiratory syndrome virus and Mycoplasma hyopneumoniae. Journal of Swine Health and Production. 2011;19(5):292-4. PubMed PMID: WOS:000294585800007.
31. Dee S, Torremorell M, Thompson B, Deen J, Pijoan C. An evaluation of thermo-assisted drying and decontamination for the elimination of porcine reproductive and respiratory syndrome virus from contaminated livestock transport vehicles. Canadian Journal of Veterinary Research-Revue Canadienne De Recherche Veterinaire. 2005;69(1):58-63. PubMed PMID: WOS:000226958100009.
32. Dimmich MM, Delks AM, Amass SF, Sheridan M. Duration of viability of desiccated porcine reproductive and respiratory syndrome virus in broken vials of Ingelvac (R) PRRS modified live vaccine. Journal of Swine Health and Production. 2006;14(5):265-6. PubMed PMID: WOS:000240077200008.
33. Eisenloffel L, Reutter T, Horn M, Schlegel S, Truyen U, Speck S. Impact of UVC-sustained recirculating air filtration on airborne bacteria and dust in a pig facility. Plos One. 2019;14(11):18. doi: 10.1371/journal.pone.0225047. PubMed PMID: WOS:000532694400092.
34. Eisenlöffel L, Reutter T, Horn M, Schlegel S, Truyen U, Speck S. Impact of UVC-sustained recirculating air filtration on airborne bacteria and dust in a pig facility. PLoS ONE. 2019;14(11). doi: 10.1371/journal.pone.0225047.
35. Eisenloffel L, Reutter T, Horn M, Schlegel S, Truyen U, Speck S. Impact of UVC-sustained recirculating air filtration on airborne bacteria and dust in a pig facility. Plos One. 2019;14(11):18. doi: 10.1371/journal.pone.0225047. PubMed PMID: WOS:000532694400092.
36. Evans CM, Medley GF, Green LE. Porcine reproductive and respiratory syndrome virus (PRRSV) in GB pig herds: farm characteristics associated with heterogeneity in

seroprevalence. Bmc Veterinary Research. 2008;4. doi: 10.1186/1746-6148-4-48. PubMed PMID: WOS:000262310300001.

37. Fano E, Pijoan C, Dee S. Evaluation of the aerosol transmission of a mixed infection of Mycoplasma hyopneumoniae and porcine reproductive and respiratory syndrome virus. Veterinary Record. 2005;157(4):105-8. doi: 10.1136/vr.157.4.105.
38. Frydas IS, Nauwynck HJ. Replication characteristics of eight virulent and two attenuated genotype 1 and 2 porcine reproductive and respiratory syndrome virus (PRRSV) strains in nasal mucosa explants. Veterinary Microbiology. 2016;182:156-62. doi: 10.1016/j.vetmic.2015.11.016.
39. Haredasht SA, Polson D, Main R, Lee K, Holtkamp D, Martinez-Lopez B. Modeling the spatio-temporal dynamics of porcine reproductive & respiratory syndrome cases at farm level using geographical distance and pig trade network matrices. Bmc Veterinary Research. 2017;13. doi: 10.1186/s12917-017-1076-6. PubMed PMID: WOS:000402977600001.
40. Hennig-Pauka I, Verspohl J, Wohlsein P, Waldmann KH, Wendt M. Multifactorial respiratory tract disease in fattening pigs in an outside climate house. Praktische Tierarzt. 2012;93(10):928-+. PubMed PMID: WOS:000309986700006.
41. Hermann JR, Brockmeier SL, Yoon KJ, Zimmerman JJ. Detection of respiratory pathogens in air samples from acutely infected pigs. Canadian Journal of Veterinary Research-Revue Canadienne De Recherche Veterinaire. 2008;72(4):367-70. PubMed PMID: WOS:000257974000011.
42. Hermann JR, Hoff SJ, Yoon KJ, Burkhardt AC, Evans RB, Zimmerman JJ. Optimization of a sampling system for recovery and detection of airborne porcine reproductive and respiratory syndrome virus and swine influenza virus. Applied and Environmental Microbiology. 2006;72(7):4811-8. doi: 10.1128/aem.00472-06. PubMed PMID: WOS:000238961000039.
43. Hermann JR, Muñoz-Zanzi CA, Zimmerman JJ. A method to provide improved dose-response estimates for airborne pathogens in animals: An example using porcine

reproductive and respiratory syndrome virus. Veterinary Microbiology. 2009;133(3):297-302. doi: 10.1016/j.vetmic.2008.07.002.

44. Hermann JR, Zimmerman JJ. Analytical sensitivity of air samplers based on uniform point-source exposure to airborne Porcine reproductive and respiratory syndrome virus and swine influenza virus. Canadian Journal of Veterinary Research-Revue Canadienne De Recherche Veterinaire. 2008;72(5):440-3. PubMed PMID: WOS:000260451600010.
45. Hermann J, Hoff S, Munoz-Zanzi C, Yoon KJ, Roof M, Burkhardt A, et al. Effect of temperature and relative humidity on the stability of infectious porcine reproductive and respiratory syndrome virus in aerosols. Veterinary Research. 2007;38(1):81-93. doi: 10.1051/vetres:2006044. PubMed PMID: WOS:000244209200003.
46. Janni KA, Torremorell M, Jacobson LD, Alonso C, Hetchler BP. Modeling airborne virus concentration in filtered swine barns with negative-pressure ventilating systems. Transactions of the Asabe. 2018;61(3):1089-99. doi: 10.13031/trans.12561. PubMed PMID: WOS:000436494500028.
47. Kauffold J, Beckjunker J, Scheller R, Schwarz BA, Beynon N, Sobiraj A. Effects of type of machine and covering on viruses and microorganisms recovered from dust in ultrasound machines used in German swine production operations. Journal of Swine Health and Production. 2005;13(2):72-80. PubMed PMID: WOS:000227408300005.
48. Kauffold J, Wehrend A, Schwarz BA, Knauf D, Willig R, Schagemann G, et al. Exhaust expulsion of the porcine reproductive and respiratory syndrome virus (PRRSV) through ultrasound machines. Tieraerztliche Praxis Ausgabe Grosstiere Nutztiere. 2010;38(5):285-92. PubMed PMID: WOS:000284730300003.
49. Kristensen CS, Bower A, Takai H, Nielsen JP, Jorsal SE. Experimental airborne transmission of PRRS virus. Veterinary Microbiology. 2004;99(3-4):197-202. doi: 10.1016/j.vetmic.2004.01.005. PubMed PMID: WOS:000220733600005.
50. Kwon HJ, Lee CH, Choi EJ, Song JY, Heinze BC, Yoon JY. Optofluidic device monitoring and fluid dynamics simulation for the spread of viral pathogens in a livestock environment. Journal of Environmental Monitoring. 2010;12(11):2138-44. doi: 10.1039/c0em00365d. PubMed PMID: WOS:000283935000017.

51. La A, Zhang Q, Cicek N, Levin DB, Coombs KM. Dose-response modelling of infectious animal diseases coupled with computational fluid dynamics: A simulation of airborne porcine reproductive and respiratory syndrome virus. Biosystems Engineering. 2021;208:58-78. doi: 10.1016/j.biosystemseng.2021.05.005. PubMed PMID: WOS:000671437900005.
52. La A, Zhang Q, Levin DB, Coombs KM. The effectiveness of air ionization in reducing bio aerosols and airborne PRRS virus in a ventilated space. Transactions of the Asabe. 2019;62(5):1299-314. doi: 10.13031/trans.13430. PubMed PMID: WOS:000510170700025.
53. Lambert ME, Arsenault J, Poljak Z, D'Allaire S. Epidemiological investigations in regard to porcine reproductive and respiratory syndrome (PRRS) in Quebec, Canada. Part 2: Prevalence and risk factors in breeding sites. Preventive Veterinary Medicine. 2012;104(1-2):84-93. doi: 10.1016/j.prevetmed.2011.11.002. PubMed PMID: WOS:000301307100009.
54. Lambert ME, Poljak Z, Arsenault J, D'Allaire S. Epidemiological investigations in regard to porcine reproductive and respiratory syndrome (PRRS) in Quebec, Canada. Part 1: Biosecurity practices and their geographical distribution in two areas of different swine density. Preventive Veterinary Medicine. 2012;104(1-2):74-83. doi: 10.1016/j.prevetmed.2011.12.004. PubMed PMID: WOS:000301307100008.
55. Larochelle R, D'Allaire S, Magar R. Molecular epidemiology of porcine reproductive and respiratory syndrome virus (PRRSV) in Quebec. Virus Research. 2003;96(1-2):3-14. doi: 10.1016/s0168-1702(03)00168-0. PubMed PMID: WOS:000185447300001.
56. Li H, Wei XB, Zhang XL, Xu H, Zhao XS, Zhou SF, et al. Establishment of a multiplex RT-PCR assay for identification of atmospheric virus contamination in pig farms. Environmental Pollution. 2019;253:358-64. doi: 10.1016/j.envpol.2019.07.028. PubMed PMID: WOS:000483406700038.
57. Li PY, Koziel JA, Zimmerman JJ, Hoff SJ, Zhang JQ, Cheng TY, et al. Designing and Testing of a System for Aerosolization and Recovery of Viable Porcine Reproductive and Respiratory Syndrome Virus (PRRSV): Theoretical and Engineering Considerations.

Frontiers in Bioengineering and Biotechnology. 2021;9:13. doi: 10.3389/fbioe.2021.659609. PubMed PMID: WOS:000653139900001.
58. Li PY, Koziel JA, Zimmerman JJ, Zhang JQ, Cheng TY, Yim-Im W, et al. Mitigation of Airborne PRRSV Transmission with UV Light Treatment: Proof-of-Concept. Agriculture-Basel. 2021;11(3):20. doi: 10.3390/agriculture11030259. PubMed PMID: WOS:000633207200001.
59. Li P, Koziel JA, Zimmerman JJ, Hoff SJ, Zhang J, Cheng TY, et al. Designing and Testing of a System for Aerosolization and Recovery of Viable Porcine Reproductive and Respiratory Syndrome Virus (PRRSV): Theoretical and Engineering Considerations. Frontiers in Bioengineering and Biotechnology. 2021;9. doi: 10.3389/fbioe.2021.659609.
60. Li P, Koziel JA, Zimmerman JJ, Hoff SJ, Zhang J, Cheng TY, et al., editors. Method for aerosolization and collection of Porcine Reproductive and Respiratory Syndrome Virus (PRRSV): Engineering considerations. American Society of Agricultural and Biological Engineers Annual International Meeting, ASABE 2021; 2021.
61. Linhares DCL, Cano JP, Wetzell T, Nerem J, Torremorell M, Dee SA. Effect of modified-live porcine reproductive and respiratory syndrome virus (PRRSv) vaccine on the shedding of wild-type virus from an infected population of growing pigs. Vaccine. 2012;30(2):407-13. doi: 10.1016/j.vaccine.2011.10.075. PubMed PMID: WOS:000299971800042.
62. Mondaca-Fernandez E, Morrison RB. Applying spatial analysis to a porcine reproductive and respiratory syndrome regional control programme. Veterinary Record. 2007;161(4):137-8. doi: 10.1136/vr.161.4.137. PubMed PMID: WOS:000248643500012.
63. More S, Botner A, Butterworth A, Calistri P, Depner K, Edwards S, et al. Assessment of listing and categorisation of animal diseases within the framework of the Animal Health Law (Regulation (EU) No2016/429): porcine reproductive and respiratory syndrome (PRRS). Efsa Journal. 2017;15(7):34. doi: 10.2903/j.efsa.2017.4949. PubMed PMID: WOS:000406685200067.
64. Mousing J, Permin A, Mortensen S, Botner A, Willeberg P. A case-control questionnaire survey of risk factors for porcine reproductive and respiratory syndrome (PRRS)

seropositivity in Danish swine herds. Veterinary Microbiology. 1997;55(1-4):323-8. doi: 10.1016/s0378-1135(96)01321-1. PubMed PMID: WOS:A1997XH36000039.

65. Mukherjee P, Karam A, Singh U, Chakraborty AK, Huidrom S, Sen A, et al. Seroprevalence of selected viral pathogens in pigs reared in organized farms of Meghalaya from 2014 to 16. Veterinary World. 2018;11(1):42-7. doi: 10.14202/vetworld.2018.42-47.
66. Nayak G, Andrews AJ, Marabella I, Aboubakr HA, Goyal SM, Olson BA, et al. Rapid inactivation of airborne porcine reproductive and respiratory syndrome virus using an atmospheric pressure air plasma. Plasma Processes and Polymers. 2020;17(10):15. doi: 10.1002/ppap.201900269. PubMed PMID: WOS:000515150900001.
67. Niederwerder MC, Rowland RRR. Is There a Risk for Introducing Porcine Reproductive and Respiratory Syndrome Virus (PRRSV) Through the Legal Importation of Pork? Food and Environmental Virology. 2017;9(1). doi: 10.1007/s12560-016-9259-z.
68. Nirmala J, Alves G, Vilalta C, Yang M, Rendahl A, Olson B, et al. Evaluation of viral RNA extraction methods to detect porcine reproductive and respiratory syndrome and influenza A viruses from used commercial HVAC air filters from swine farms. Journal of Aerosol Science. 2021;151:12. doi: 10.1016/j.jaerosci.2020.105624. PubMed PMID: WOS:000591603000007.
69. Otake S, Dee SA, Jacobson L, Torremorell M, Pijoan C. Evaluation of aerosol transmission of porcine reproductive and respiratory syndrome virus under controlled field conditions. Veterinary Record. 2002;150(26):804-8. doi: 10.1136/vr.150.26.804.
70. Otake S, Dee S, Corzo C, Oliveira S, Deen J. Long-distance airborne transport of infectious PRRSV and Mycoplasma hyopneumoniae from a swine population infected with multiple viral variants. Veterinary Microbiology. 2010;145(3-4):198-208. doi: 10.1016/j.vetmic.2010.03.028. PubMed PMID: WOS:000283685200002.
71. Perez AM, Davies PR, Goodell CK, Holtkamp DJ, Mondaca-Fernandez E, Poljak Z, et al. Lessons learned and knowledge gaps about the epidemiology and control of porcine reproductive and respiratory syndrome virus in North America. Javma-Journal of the American Veterinary Medical Association. 2015;246(12):1304-17. doi: 10.2460/javma.246.12.1304. PubMed PMID: WOS:000359850100025.

72. Pileri E, Mateu E. Review on the transmission porcine reproductive and respiratory syndrome virus between pigs and farms and impact on vaccination. Veterinary Research. 2016;47:13. doi: 10.1186/s13567-016-0391-4. PubMed PMID: WOS:000386737300001.
73. Pitkin A, Deen J, Dee S. Use of a production region model to assess the airborne spread of porcine reproductive and respiratory syndrome virus. Veterinary Microbiology. 2009;136(1-2):1-7. doi: 10.1016/j.vetmic.2008.10.013. PubMed PMID: WOS:000265431500001.
74. Prieto C, Castro JM. Porcine reproductive and respiratory syndrome virus infection in the boar: a review. Theriogenology. 2005;63(1):1-16. doi: 10.1016/j.theriogenology.2004.03.018. PubMed PMID: WOS:000227694300001.
75. Reicks DL. Effective biosecurity to protect North American studs and clients from emerging infectious disease. Theriogenology. 2019;137:82-7. doi: 10.1016/j.theriogenology.2019.05.041. PubMed PMID: WOS:000477691200013.
76. Rosendal T, Dewey C, Friendship R, Wootton S, Young B, Poljak Z. Spatial and temporal patterns of porcine reproductive and respiratory syndrome virus (PRRSV) genotypes in Ontario, Canada, 2004-2007. Bmc Veterinary Research. 2014;10. doi: 10.1186/1746-6148-10-83. PubMed PMID: WOS:000335184200001.
77. Sanhueza JM, Stevenson MA, Vilalta C, Kikuti M, Corzo CA. Spatial relative risk and factors associated with porcine reproductive and respiratory syndrome outbreaks in United States breeding herds. Preventive Veterinary Medicine. 2020;183:9. doi: 10.1016/j.prevetmed.2020.105128. PubMed PMID: WOS:000579919400019.
78. Schelkopf A, Nerem J, Cowles B, Amodie D, Swalla R, Dee S. Reproductive, productivity, and mortality outcomes in late-gestation gilts and their litters following simulation of inadvertent exposure to a modified-live vaccine strain of porcine reproductive and respiratory syndrome (PRRS) virus. Vaccine. 2014;32(36):4639-43. doi: 10.1016/j.vaccine.2014.06.073. PubMed PMID: WOS:000340326600007.
79. Smith B, Hoff S, Harmon J, Andersen D, Zimmerman J, Stinn J. Quantification of Site Layout and Filter Characteristics on Primary Filter Airflow Reduction on Commercial

Swine Sites in Iowa. Agriengineering. 2019;1(2):291-302. doi: 10.3390/agriengineering1020022. PubMed PMID: WOS:000661678900001.

80. Spronk G, Otake S, Dee S. Prevention of PRRSV infection in large breeding herds using air filtration. Veterinary Record. 2010;166(24):758-9. doi: 10.1136/vr.b4848. PubMed PMID: WOS:000279239300021.
81. Stein H, Schulz J, Morgenstern R, Voglmayr T, Freymuller G, Sinn L, et al. Use of Three Air Samplers for the Detection of PRRSV-1 under Experimental and Field Conditions. Animals. 2018;8(12):6. doi: 10.3390/ani8120233. PubMed PMID: WOS:000454708000015.
82. Torremorell M, Pijoan C, Janni K, Walker R, Joo HS. Airborne transmission of Actinobacillus pleuropneumoniae and porcine reproductive and respiratory syndrome virus in nursery pigs. American Journal of Veterinary Research. 1997;58(8):828-32. PubMed PMID: WOS:A1997XN78400010.
83. Tousignant SJP, Perez AM, Lowe JF, Yeske PE, Morrison RB. Temporal and spatial dynamics of porcine reproductive and respiratory syndrome virus infection in the United States. American Journal of Veterinary Research. 2015;76(1):70-6. doi: 10.2460/ajvr.76.1.70. PubMed PMID: WOS:000351422900007.
84. Trincado C, Dee S, Jacobson L, Otake S, Rossow K, Pijoan C. Attempts to transmit porcine reproductive and respiratory syndrome virus by aerosols under controlled field conditions. Veterinary Record. 2004;154(10):294-7. doi: 10.1136/vr.154.10.294. PubMed PMID: WOS:000220379000010.
85. Velasova M, Alarcon P, Williamson S, Wieland B. Risk factors for porcine reproductive and respiratory syndrome virus infection and resulting challenges for effective disease surveillance. Bmc Veterinary Research. 2012;8. doi: 10.1186/1746-6148-8-184. PubMed PMID: WOS:000315721100001.
86. Vilalta C, Sanhueza J, Garrido J, Murray D, Morrison R, Corzo CA, et al. Indirect assessment of porcine reproductive and respiratory syndrome virus status in pigs prior to weaning by sampling sows and the environment. Veterinary Microbiology.

2019;237:7. doi: 10.1016/j.vetmic.2019.108406. PubMed PMID: WOS:000491213800008.

87. Wenke C, Pospiech J, Reutter T, Truyen U, Speck S. Efficiency of different air filter types for pig facilities at laboratory scale. Plos One. 2017;12(10):12. doi: 10.1371/journal.pone.0186558. PubMed PMID: WOS:000412980300041.

88. Wills RW, Zimmerman JJ, Swenson SL, Yoon KJ, Hill HT, Bundy DS, et al. Transmission of PRRSV by direct, close, or indirect contact. Journal of Swine Health and Production. 1997;5(6):213-8.

89. Xia T, Yang M, Marabella I, Lee EM, Olson B, Zarling D, et al. Inactivation of airborne porcine reproductive and respiratory syndrome virus (PRRSv) by a packed bed dielectric barrier discharge non-thermal plasma. Journal of Hazardous Materials. 2020;393:8. doi: 10.1016/j.jhazmat.2020.122266. PubMed PMID: WOS:000532829200006.

90. Yun SI, Lee YM. Overview: Replication of porcine reproductive and respiratory syndrome virus. Journal of Microbiology. 2013;51(6):711-23. doi: 10.1007/s12275-013-3431-z. PubMed PMID: WOS:000329238900001.

91. Zhai SL, Lu SS, Wei WK, Lv DH, Wen XH, Zhai Q, et al. Reservoirs of Porcine Circoviruses: A Mini Review. Frontiers in Veterinary Science. 2019;6:6. doi: 10.3389/fvets.2019.00319. PubMed PMID: WOS:000486684700001.

92. Zhang RZ, Tsai SS, Chao HR, Shieh YC, Chuang KP. Innovation of a New Virus-Like Nanoparticle Vaccine System for the Specific Aerosol Relative Disease. Aerosol and Air Quality Research. 2016;16(10):2421-7. doi: 10.4209/aaqr.2016.09.0379. PubMed PMID: WOS:000392304700010.
